# Modeling the Arrows of Time with Causal Multibaker Maps

**DOI:** 10.3390/e26090776

**Published:** 2024-09-10

**Authors:** Aram Ebtekar, Marcus Hutter

**Affiliations:** 1 Independent Researcher, Vancouver, BC V5Y 3J6, Canada; 2 Google DeepMind, London N1C 4AG, UK; 3 School of Computing, Australian National University, Canberra, ACT 2601, Australia

**Keywords:** local causality, Markov property, memory systems, psychological arrow of time, records, second law of thermodynamics, symbolic dynamics

## Abstract

Why do we remember the past, and plan the future? We introduce a toy model in which to investigate emergent time asymmetries: the causal multibaker maps. These are reversible discrete-time dynamical systems with configurable causal interactions. Imposing a suitable initial condition or “Past Hypothesis”, and then coarse-graining, yields a Pearlean locally causal structure. While it is more common to speculate that the other arrows of time arise from the thermodynamic arrow, our model instead takes the causal arrow as fundamental. From it, we obtain the thermodynamic and epistemic arrows of time. The epistemic arrow concerns records, which we define to be systems that encode the state of another system at another time, regardless of the latter system’s dynamics. Such records exist of the past, but not of the future. We close with informal discussions of the evolutionary and agential arrows of time, and their relevance to decision theory.

## 1. Introduction

The “arrows of time” are natural phenomena that are asymmetric under time reversal. They are responsible for the qualitative differences between how we relate to the past and the future, governing our experience of the irreversible passage of time.

To review some important examples, the **thermodynamic arrow of time** is the observation that entropy increases over time [1,2].

The **causal arrow of time** is the observation that causes precede effects [3,4]. It is a key assumption in Bell’s inequality [5,6], and in the design and interpretation of scientific experiments more generally [7,8,9,10].

The **epistemic arrow of time** is the observation that there are records of past events, but not of future events [3,4,11,12]. While some authors refer to it as a “psychological arrow” [13,14], the epistemic arrow is not restricted to biological memories: footprints, fossils, impact craters, and photographs all serve as records of past events.

We find other irreversible phenomena in complex, living systems. The **evolutionary arrow of time** is the observation that Darwinian natural selection makes each successive generation better-adapted to its environment [15,16]. At first glance, the evolution of “order” (in the sense of adaptation) seems contrary to the thermodynamic trend toward “disorder” (in the sense of entropy). While the release of entropy into the environment by metabolic processes ensures that there is no contradiction [17], one can still ask why the arrows are aligned.

Finally, the **agential/volitional arrow of time** refers to the observation that intelligent agents model their past as “fixed”, whereas their future is “open” to influence from their present choice of action [3,4,18]. We plan our future retirement, not our past childhood.

The arrows of time are so intertwined, that one may wonder if they are really one and the same. For example, the genes we obtain from Darwinian evolution encode traits that were adaptive in our ancestors’ environment, serving as an epistemic record of past generations. Moreover, evolution produces intelligent agents, who choose a “best” action according to a form of causal reasoning that compares the counterfactual outcomes of different actions [9,19,20]. The past’s immunity to counterfactual change is reminiscent of the robustness of epistemic records, while the future’s epistemic uncertainty is reminiscent of entropy.

Unfortunately, there is not yet any rigorous and general derivation of the arrows of time from fundamental physics. In physical theories with an *initial value formulation*, the Universe is fully determined by its *initial condition* and *dynamical laws* [21]. In this article, we assume that such a formulation exists. Causal reasoning then appears to pose a paradox: interventions and counterfactuals, in describing alternative futures, would violate both determinism and time-reversal symmetry [20,22].

Indeed, the dynamical laws of physics are widely believed to be time-reversal symmetric (more precisely, CPT symmetric [23]). The only remaining piece of our physics formulation, which might produce the needed asymmetry, is its initial condition. It is important to bear in mind that we can equally well determine the Universe by evolving its dynamics backward, from a *final condition* at any later time (i.e., Cauchy surface [21]). Therefore, in order to truly break the symmetry, the initial condition must somehow be special in comparison to later states of the Universe.

The **Past Hypothesis** is the idea that special conditions at the Big Bang explain the arrows of time that we experience today [2,24,25]. A rigorous argument from the Past Hypothesis remains out of reach [12], at least for real systems evolving according to classical or quantum Hamiltonians.

On the other hand, recent advances in Markovian **stochastic thermodynamics** provide a powerful framework for studying irreversible phenomena [26,27,28,29]. The idea is to model classical phase space trajectories at a fixed level of precision. The result is a stochastic process over a coarse-grained set of states, each representing some small region in phase space (as in [1] Section 12, or [28] Section 2.7).

We assume this process to be Markovian. While the property of being a Markov chain is invariant under time-reversal, many properties of the Markov kernel are not, such as: homogeneity, locality, algorithmic complexity, and stationarity with respect to a coarse-grained Liouville measure [30,31,32,33]. For instance, consider the time-reversed trajectory of a shattered vase: distant shards begin to rise together at the same time, seemingly on their own. At the coarse-grained level, where molecular collisions are hidden, this phenomenon appears nonlocal.

Within the Markovian framework, the thermodynamic arrow is easily shown [27,28,29]. There is a large literature studying conditions under which the Markov property holds approximately [34,35,36,37,38]; however, these are fairly complicated, obfuscating their connection to the non-thermodynamic arrows of time. Instead, we examine coarse-grainings of simpler toy systems in discrete time, for which the Markov property holds *exactly*.

Gaspard [39] introduces such a system, the *multibaker map*, to study how deterministic and reversible microscopic dynamics can, upon taking a coarse-grained view, emulate diffusive random walks, leading to an irreversible increase in entropy. Altaner and Vollmer [40] introduce an extension, the *network multibaker maps*, to emulate general Markov chains. These maps serve as tractable examples of reversible dynamical systems, on which the theorems of stochastic thermodynamics apply. As such, we can study the relationship between their fine-grained and coarse-grained properties, in the hopes that some insights will carry over to the real Universe.

The Past Hypothesis for a multibaker map is its initial distribution. In [39,40], the fine-grained microscopic details initially are uniformly distributed. An unpublished manuscript [33] relaxes the initialization requirements, by showing that any continuous initial distribution eventually renders the coarse-grained dynamics approximately Markovian, thereby increasing entropy.

While the multibaker maps can model the thermodynamic arrow, the other arrows of time require us to model interactions between multiple physical systems. The reason is that memory systems need an external system to record, and agents need an environment with which to interact.

Pearl [9] generalizes the Markov property from mere chains to directed acyclic graphs, which model interacting systems. Moreover, he augments the graphs with the *causal* semantics of interventions and counterfactuals. The history of causal concepts spans the disciplines of philosophy, physics, statistics, computer science, and economics, with important contributions from Neyman [41], Reichenbach [3], Lewis [20,42], Bell [5,6], Granger [7,43], Holland [44], Imbens and Rubin [8], and Spirtes et al. [45]. We focus on Pearl [9] due to his convenient structural semantics, which have already been applied to stochastic thermodynamics by Ito and Sagawa [46,47], and Wolpert [48].

In this article, we introduce **causal multibaker maps**. Just as network multibaker maps emulate Markov chains upon coarse-graining, causal multibaker maps emulate Pearlean causal models. In the resulting models, we demonstrate that entropy cannot decrease, and future events cannot be reliably recorded. Thus, we take the causal arrow as fundamental, and use it to explain the thermodynamic, epistemic, evolutionary, and agential arrows. This approach is in line with Markovian stochastic thermodynamics, which uses the Markov assumption to prove thermodynamic relations, but departs from prior efforts to explain the arrows of time, which instead take the thermodynamic arrow as fundamental [3,4,11,12,14,18,49].

Compared to these works, ours has the advantage of being mathematically precise, and applying to a wider variety of coarse-grained dynamics. The drawback is that our underlying microscopic dynamics are of the multibaker type. As such, we can make no definitive conclusions about the arrows of time in the real physical Universe, leaving such extensions to future work.

Nonetheless, to our knowledge, our causal multibaker maps are the first deterministic time-reversible models, that are rigorously shown to possess an emergent causal and epistemic arrow of time at the coarse-grained level. We propose them as a testbed in which to refine hypotheses about the Universe’s time asymmetries.

Finally, we point to some related models. *Symbolic dynamics* is a mathematical discipline that studies chaos and irreversibility, using simplified models similar to the multibaker maps [50,51]. Since our models feature subsystems at discrete sites, evolving in discrete time, they can also be considered a kind of *coupled map lattice* [52].

The unpublished manuscript [33] discusses a cellular automaton extension of the multibaker maps. The automaton’s dynamical homogeneity in space and time mimics that of field equations in physics. Like field theories, cellular automata propagate influences at the “speed of light”, making it difficult to causally insulate subsystems from one another [53]. In order to more easily model periods of non-interaction, our causal multibaker maps allow non-homogeneity.

*Article outline.* Section 2 sets up some notation, as well as definitions of information-theoretic quantities. Section 3 first develops causal models, and then their microscopic description in terms of causal multibaker maps. In this context, Section 4 discusses each of the arrows of time in turn. Finally, Section 5 discusses future research directions.

## 2. Preliminaries

We start with some notation. Z and R denote the integers and real numbers, respectively, while $Z_{m}:=\left\{0,1,\ldots ,m-1\right\}$ denotes the first *m* non-negative integers. ∅ is the empty set, and $\delta_{x,\;x^{\prime }}$ is the Kronecker delta that equals 1 if $x=x^{\prime }$, and 0 otherwise.

When a capital letter such as *X* refers to a random variable, its lowercase counterparts $x,x^{\prime }$ refer to the specific values it takes on. This allows us to write Pr(x∣y) as shorthand for the conditional probability expression Pr(X=x∣Y=y).

In classical information theory [54], the **Shannon entropy** of a random variable *X* is

$$H\left(X\right):=\sum_{x}\mathrm{Pr}\left(x\right)\log\frac{1}{\mathrm{Pr}(x)}.$$

The **conditional Shannon entropy** of *X*, given another random variable *Y*, is

$$H\left(X|Y\right):=H\left(X,\;Y\right)-H\left(Y\right)=\sum_{x,\;y}\mathrm{Pr}\left(x,\;y\right)\log\frac{1}{\mathrm{Pr}(x|y)}.$$

Finally, the **mutual information** between *X* and *Y* is

I(X:Y):=H(X)+H(Y)−H(X,Y)=H(X)−H(X∣Y)=H(Y)−H(Y∣X).

## 3. Causal Dynamics

This section consists of four parts, each describing one kind of model. First, we review general causal models on directed acyclic graphs, summarizing some of the core insights from Pearl [9].

Then, we specialize the models to a coarse-grained (mesoscopic) view of causally interacting physical subsystems. We call this view a **persistent causal model**, since each subsystem has an identity that persists over time, its trajectory being represented by a sequence of vertices in the graph. Similar models appear in the stochastic thermodynamics literature [46,47,48]; since thermodynamic concepts such as heat and work do not enter our discussion, our definitions are a bit simpler. Unlike dynamic Bayesian networks that change over time [55,56,57], we use a fixed graph to model the states at all times.

To each persistent causal model with doubly stochastic transition probabilities, we associate a **dual model** whose edges point in the opposite temporal direction.

The previous models all have Pearl’s causal arrow of time baked in. In order to connect them to time-symmetric dynamics, we finally present a fine-grained (microscopic) view of persistent causal models: the **causal multibaker maps**. Its dynamics are deterministic and reversible. Therefore, the asymmetry that emerges in the coarse-grained view can be traced back to special properties of the fine-grained initial condition (i.e., a Past Hypothesis).

### 3.1. Review of Causal Models

Consider a finite set of random variables $V=\{V_{1},V_{2},\ldots ,V_{n}\}$, which we identify with the *n* vertices of a directed acyclic graph. Thus, each $V_{i}\in V$ is both a random variable and a vertex. Its **parents**, or **direct causes**, are the random variables $PA_{i}\subset V$ that have an outgoing edge directly to $V_{i}$. Since the sets $PA_{i}$ together determine all the edges, we can identify the graph with the pair G:=(V,PA).

We say the random variables V satisfy the **Markov property**, or are **Markovian**, with respect to the graph G, if their joint distribution factorizes as

(1) $$\mathrm{Pr}\left(v\right)=\prod_{i=1}^{n}\mathrm{Pr}\left(v_{i}|pa_{i}\right).$$

In the special case where $PA_{1}=\emptyset$ and $PA_{i}=\left\{V_{i-1}\right\}$ for i>1, V is called a **Markov chain**.

The Markov property (1) has a useful graphical characterization. For any disjoint sets of variables X,Y,Z⊂V, we say that *X* and *Y* are **d-connected**, given *Z*, if G has a path $\left(V_{p(0)},\;V_{p(1)}\;\ldots ,\;V_{p(m)}\right)$ such that:

- $V_{p(0)}\in X$.
- $V_{p(m)}\in Y$.
- For 0<i<m, if $V_{p(i)}\notin Z$, then $V_{p(i)}$ is a *chain* or a *fork* on the path, i.e., the edges are oriented as either $V_{p(i-1)}\to V_{p(i)}\to V_{p(i+1)}$, $V_{p(i-1)}\leftarrow V_{p(i)}\leftarrow V_{p(i+1)}$, or $V_{p(i-1)}\leftarrow V_{p(i)}\to V_{p(i+1)}$.
- For 0<i<m, if $V_{p(i)}\in Z$, then $V_{p(i)}$ is a *collider* on the path, i.e., the edges are oriented as $V_{p(i-1)}\to V_{p(i)}\leftarrow V_{p(i+1)}$.

We say that *X* and *Y* are **d-separated**, given *Z*, if they are not d-connected. Verma and Pearl [58] prove the **d-separation criterion**: V is Markovian with respect to G iff for all disjoint X,Y,Z⊂V, d-separation of *X* and *Y* given *Z* implies conditional independence of *X* and *Y* given *Z*. Note that when *Z* is empty, d-connecting paths are always of the form $V_{p(0)}\leftarrow \cdots \leftarrow V_{p(i)}\to \cdots \to V_{p(m)}$. Therefore, if *X* and *Y* are unconditionally dependent, they must share a common ancestor $V_{p(i)}$ in G, also called a **common cause**.

We should take care not to use the term “cause” loosely. The graph G is called a **Bayesian network**, if it is only used to convey *probabilistic* information: namely, that V satisfies its Markov property (1). G is instead called a **causal network** if, in addition to the Markov property, it also conveys *causal* information about how this distribution would be modified by structural **interventions**. A Bayesian or causal **model** is the combination of a Bayesian or causal network G, together with all the factors or **mechanisms**$\mathrm{Pr}(v_{i}|pa_{i})$ that complete the right-hand side of (1).

Pearl [59] defines the intervention operator $do(V_{i}=x)$, which sets $\mathrm{Pr}\left(v_{i}|pa_{i}\right):=\delta_{v_{i},\;x}$, effectively disconnecting *v* from its parents. The remaining factors in (1) are left unchanged. In the deterministic case, where V takes on a fixed value, Bayesian models are trivial: regardless of the edges PA, there always exists a factorization of the form (1), with $\mathrm{Pr}\left(v_{i}|pa_{i}\right):=\delta_{v_{i},\;f_{i}\left(pa_{i}\right)}$ for some functions $f_{i}$. In contrast, *causal* models convey nontrivial information even in the deterministic case: the intervention $do(V_{i}=x)$ alters each descendant $V_{j}$ of $V_{i}$, according to the functions $f_{k}$ on the paths between them.

With a little more work, Pearl [9] goes on to define the **counterfactual** variables $V^{do(V_{i}=x)}$, whose distribution is given by the corresponding intervention $do(V_{i}=x)$ on V. It represents the state that our world V *would have* taken on, *had* the intervention $do(V_{i}=x)$ been performed. Notice the time-reversal asymmetry: since $V_{i}$ is cut off from its past (i.e., ancestors in G) but not its future (i.e., descendants in G), only the future is subject to change. Thus, causes precede their effects.

The philosophical and physical meaning of interventions and counterfactuals is hotly debated [9,20,25]. They certainly seem important for everyday reasoning: for example, in criminal justice, the accused might be held responsible if their action produced a less favorable outcome for the victim, compared to the counterfactual result of some alternative action. Thus, we entertain an alternative reality in which the accused behaves differently, perhaps even in a manner inconsistent with their psychological nature, which may well be a function of genetics and upbringing. In other words, a counterfactual refers to something that does not occur and may in fact violate the laws of physics [22].

This is sometimes framed as a paradox of “free will” [22], though we prefer to avoid this ambiguous term. Instead, we think of the accused as a being who has evolved a brain, which follows an algorithm to decide an action. One particularly successful kind of algorithm, which Darwinian evolution might plausibly select for, models many available actions as interventions, estimates their outcomes, and then performs the action whose outcome is most desirable to the agent. Thus, although actions are ultimately predetermined, the most successful agents tend to be ones who consider multiple alternatives [60].

Likewise, it is sensible for our systems of ethics and justice to consider counterfactuals, if their purpose is to encourage or deter the actions of citizens. In the scientific disciplines of evolutionary biology, economics, and artificial intelligence, it is common to model agents as optimizing future outcomes toward some objective. Again, this involves a comparison among interventions or counterfactuals [19].

**Figure 1:**
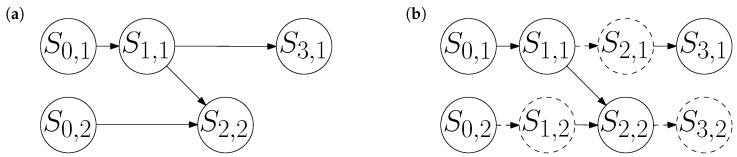
(**a**) The graph of a persistent causal model for two systems. Vertices are drawn as circles containing their variable name; edges are drawn as arrows. System 1 (top row) evolves autonomously as a Markov chain. At time t=2, it influences the external memory represented by System 2 (bottom row). (**b**) The same model, with alias variables (dashed circles) to represent non-evolving systems. These variables are exactly equal to their predecessor along a dashed edge.

In general, interventions are useful for modeling exogenous influences on an open system. For example, consider the causal network in Figure 1b. If we do not know about the variables in the top row, then we might model the bottom row as an open system, with an unknown exogenous cause acting on the endogenous variable $S_{2,\;2}$. In this setting, each possible value of $S_{1,\;1}$ may be modeled by a do operator on $S_{2,\;2}$. The interventions may even be physically realized, if the Universe contains many copies of the system represented by the bottom row, each exposed to a different exogenous context.

### 3.2. Persistent Causal Models

Now, we use causal networks to model *N* physical **systems**, numbered i=1,…,N, evolving over a sequence of *T* **events**, numbered t=1,…,T. Each system *i* is associated with a countable set $S_{i}$ of possible coarse-grained states. In general, any subset *A* of the **Universe** U:={1,…,N} may be viewed as a composite system, with associated state space $S_{A}:=\prod_{i\in A}S_{i}$. If A⊂B⊂U, we say that *A* is a **subsystem** of *B*.

Each event *t* is associated with a disjoint pair of composite systems e(t),c(t)⊂U. e(t) represents the systems that evolve during the event *t*, while all other systems are held fixed. The evolution is influenced by the systems c(t), so that the joint state probability distribution of the systems e(t) at time *t* is defined as some function of the state of the systems e(t)∪c(t) at time t−1. e(t) should be non-empty, since otherwise the event does nothing; however, c(t) can be empty.

Formally, a persistent causal model is a Pearlean causal model whose graph G has some vertices corresponding to each time t=0,1,…,T. For the initial time t=0,G has N+1 vertices:

$$\begin{array}{cccc} \; & E_{0}\in S_{U}, & \; & PA(E_{0}):=\emptyset ,\\ \mathrm{For}\;i\in U:\; & S_{0,\;i}\in S_{i}, & \; & PA\left(S_{0,\;i}\right):=\left\{E_{0}\right\}. \end{array}$$

$\mathrm{Pr}(E_{0})$ is the initial state distribution of our Universe, while each $S_{0,\;i}:={\left(E_{0}\right)}_{i}$ is deterministically assigned the initial state of system i∈U.

For each non-initial time t=1,…,T,G has |e(t)|+1 vertices:

$$\begin{array}{cccc} \; & E_{t}\in S_{e(t)}, & \; & PA\left(E_{t}\right):=\left\{S_{t-1,\;j}:\;j\in e\left(t\right)\cup c\left(t\right)\right\},\\ \mathrm{For}\;i\in e(t):\; & S_{t,\;i}\in S_{i}, & \; & PA\left(S_{t,\;i}\right):=\left\{E_{t}\right\}. \end{array}$$

In order for the expression for $PA(E_{t})$ to make sense, we define $S_{t,\;i}:=S_{t-1,\;i}$ for the non-evolving systems i∈U∖e(t); these are not additional vertices, but rather, aliases for vertices from an earlier time. $\mathrm{Pr}(E_{t}|PA\left(E_{t}\right))$ is the dynamics of event *t*, while each $S_{t,\;i}:={\left(E_{t}\right)}_{i}$ is deterministically assigned the evolved state of system i∈e(t).

For convenience, we denote the state of a composite system A⊂U at time *t* by $S_{t,\;A}:=\left(S_{t,\;i}:\;i\in A\right)$. Our causal network indicates a joint probability distribution that factorizes as (1). Since $E_{0}=S_{0,\;U}$ and $E_{t}=S_{t,\;e(t)}$ are effectively contained in the collection of random variables S, it suffices to expand the latter’s distribution:

(2) $$\mathrm{Pr}\left(s\right):=\mathrm{Pr}\left(s_{0,\;U}\right)\prod_{t=1}^{T}\mathrm{Pr}\left(s_{t,\;e(t)}|s_{t-1,\;e(t)},\;s_{t-1,\;c(t)}\right).$$

In summary, a persistent causal model is specified by its event structure ${\left(e\left(t\right),\;c\left(t\right)\right)}_{t=1}^{T}$ (which determines the graph G), its **initial condition**$\mathrm{Pr}(s_{0,\;U})$, and its events’ **forward mechanisms**$\mathrm{Pr}(s_{t,\;e(t)}|s_{t-1,\;e(t)},\;s_{t-1,\;c(t)})$. The case N=1 corresponds to a Markov chain, whereas having N>1 allows us to model interactions between *N* systems.

In cases where the systems are initially independent, we can omit the vertex $E_{0}$ and directly assign each respective initial distribution $\mathrm{Pr}(S_{0,\;i})$:

$$\begin{array}{cccc} \mathrm{For}\;i\in U:\; & S_{0,\;i}\in S_{i}, & \; & PA(S_{0,\;i}):=\emptyset . \end{array}$$

Similarly, for events *t* that evolve only one system *i*, we can omit the vertex $E_{t}$ and directly assign the forward mechanism to $\mathrm{Pr}(S_{t,\;i}|PA\left(S_{t,\;i}\right))$:

$$\begin{array}{cccc} \mathrm{For}\;\mathrm{the}\;\mathrm{unique}\;i\in e(t):\; & S_{t,\;i}\in S_{i}, & \; & PA\left(S_{t,\;i}\right):=\left\{S_{t-1,\;j}:\;j\in e\left(t\right)\cup c\left(t\right)\right\}. \end{array}$$

Thus, in the simple case where the initial states are independent and the systems evolve one at a time, G has n=N+T vertices: one for the initial state of each system i=1,…,N, and one for each event t=1,…,T. Let’s consider one such example.

**Example 1.**  *From the two-system causal network in Figure 1a, we read off e(1)=e(3)={1}, c(1)=c(3)=∅, e(2)={2}, and c(2)={1}. Suppose System 1 has an integer-valued state that evolves autonomously on a random walk: at each of the times t=1,3, it either stays still, increments, or decrements, each with probability 1/3. Moreover, suppose that at the time t=2, System 1 reversibly writes its state to an external memory, represented by System 2.*

*Rather than explicitly tabulate all of the conditional probabilities in (2), we can implicitly define each mechanism in terms of a variable assignment:*

$$\begin{array}{cc} S_{0,\;1} & :=S_{0,\;2}:=0,\\ S_{1,\;1} & :=S_{0,\;1}+\mathrm{Uniform}\left\{-1,\;0,\;1\right\},\\ S_{2,\;2} & :=S_{1,\;1}⊕S_{0,\;2},\\ S_{3,\;1} & :=S_{1,\;1}+\mathrm{Uniform}\left\{-1,\;0,\;1\right\}, \end{array}$$

*where ⊕ denotes bitwise exclusive-or (i.e., binary addition without carries, so 5⊕3=6), and Uniform indicates an independent sample with uniform probability of being each element of the enclosed set. In Figure 1b, we add the alias variables $S_{1,\;2}:=S_{0,\;2}$, $S_{2,\;1}:=S_{1,\;1}$, and $S_{3,\;2}:=S_{2,\;2}$, for the non-evolving system at each time step.*

### 3.3. The Dual of a Persistent Causal Model

In order to reverse the causal arrow, we need an additional property: an event *t* is **doubly stochastic** if, for all $y\in S_{e(t)}$ and $z\in S_{c(t)}$,

(3) $$\sum_{x\in S_{e(t)}}\mathrm{Pr}\left(S_{t,\;e(t)}=y|S_{t-1,\;e(t)}=x,\;S_{t-1,\;c(t)}=z\right)=1.$$

We then define the event’s **dual mechanism**

(4) $$\mathrm{Pr}\left({\tilde{S}}_{t-1,\;e(t)}=x|{\tilde{S}}_{t,\;e(t)}=y,\;{\tilde{S}}_{t,\;c(t)}=z\right):=\mathrm{Pr}\left(S_{t,\;e(t)}=y|S_{t-1,\;e(t)}=x,\;S_{t-1,\;c(t)}=z\right),$$

which is a valid conditional probability distribution whenever (3) holds.

Now suppose we have a persistent causal model (2), whose events are all doubly stochastic (3); and we also have a **final condition** $\mathrm{Pr}({\tilde{s}}_{T,\;U})$. Then, the dual mechanisms (4), together with the aliases ${\tilde{S}}_{t-1,\;i}:={\tilde{S}}_{t,\;i}$ for i∈U∖e(t), determine a dual collection of random variables $\tilde{S}$, whose joint probability distribution is given by

(5) $$\mathrm{Pr}\left(\tilde{s}\right):=\mathrm{Pr}\left({\tilde{s}}_{T,\;U}\right)\prod_{t=1}^{T}\mathrm{Pr}\left({\tilde{s}}_{t-1,\;e(t)}|{\tilde{s}}_{t,\;e(t)},\;{\tilde{s}}_{t,\;c(t)}\right).$$

The causal network for $\tilde{S}$ is inferred from Equation (5). Rather than describe it in abstract terms, we demonstrate with an example.

**Example 2.**  *The reader may verify that the events in Example 1 are not only doubly stochastic, but also self-dual. Therefore, its dual mechanisms are well-defined and identical to its forward mechanisms. Moreover, the sequence of events is symmetric, with System 1 taking a random step both before and after writing to System 2. If we complete the symmetry by setting the dual model’s final condition to match the forward model’s initial condition, then the two models become fully identical, except that the dual model’s time indices are reversed:*

$$\begin{array}{cc} {\tilde{S}}_{3,\;1} & :={\tilde{S}}_{3,\;2}:=0,\\ {\tilde{S}}_{2,\;1} & :={\tilde{S}}_{3,\;1}+\mathrm{Uniform}\left\{-1,\;0,\;1\right\},\\ {\tilde{S}}_{1,\;2} & :={\tilde{S}}_{2,\;1}⊕{\tilde{S}}_{3,\;2},\\ {\tilde{S}}_{0,\;1} & :={\tilde{S}}_{2,\;1}+\mathrm{Uniform}\left\{-1,\;0,\;1\right\}. \end{array}$$

**Figure 2:**
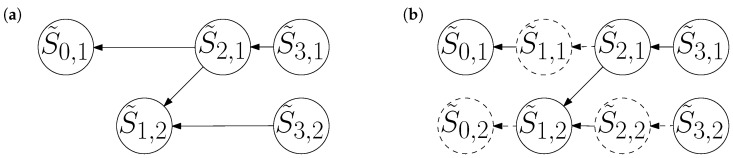
(**a**) The dual of Figure 1a; a causal network whose edges point to decreasing times. (**b**) The dual network with alias variables (dashed circles) included. Note that dashed edges appear in exactly the same positions as in Figure 1b, but they point to different dashed circles.

*The causal network for this dual process, shown in Figure 2, is a mirror image of the original network in Figure 1. However, we must remark that the dual process does not undo the forward process. That is, suppose we change the dual model’s final condition to match the forward model’s final distribution:*

$$\left({\tilde{S}}_{3,\;1},\;{\tilde{S}}_{3,\;2}\right):=\left(S_{3,\;1},\;S_{3,\;2}\right).$$

*Then, the dual process does not undo System 1’s random walk to return to $S_{0,\;1}:=0$. Instead, it takes two additional steps, making ${\tilde{S}}_{0,\;1}$ the result of four independent random steps; its value is randomly distributed over the range [−4,4].*

Intuitively speaking, the dual process corresponds to a coarse-grained approximation of time reversal, in which a loss of fine-grained correlations prevents random processes from being undone. It would be like trying to unstir a cup of coffee by reversing the mixing spoon’s trajectory. To make this idea precise, we need a fine-grained model.

### 3.4. A Microscopic View: Causal Multibaker Maps

Our fine-grained model, the **causal multibaker map**, is a multi-system extension of the traditional baker and multibaker maps [39,40]. Its dynamics are deterministic and reversible. It can be given either an initial or a final condition; accordingly, a causal multibaker map’s coarse-grained behavior is either that of the persistent causal model (2), or its dual (5), respectively.

Moreover, the correspondence between fine-grained and coarse-grained dynamics is localized to individual events. Thus, an event’s coarse-grained transition probabilities *along* the causal network’s edges can be predicted from local knowledge of the event’s fine-grained mechanism, and controlled by modifying this mechanism. In contrast, an event’s transition probabilities *against* the edges depend nonlocally on other mechanisms. This provides a causal arrow of time.

To describe a causal multibaker map, assume *N*, *T*, and ${\left(e\left(t\right),\;c\left(t\right)\right)}_{t=1}^{T}$ are given as before, and fix an integer m>1. A fine-grained **microstate** for system *i* is a pair of the form

(6) $$(x,\;\left(\ldots ,\;r_{-2},\;r_{-1},\;r_{0},\;r_{1},\;r_{2},\;\ldots \right)),$$

consisting of a coarse-grained state $x\in S_{i}$, along with a bi-infinite sequence of microvariables $r_{k}\in Z_{m}$. The full microstate of our model Universe at any given time consists of *N* such pairs, one for each system.

To get some geometric intuition, we can identify $S_{i}$ with Z, and interpret (6) as a point in the two-dimensional “phase space” R×[0,1], whose base *m* representation is

(7) $$(x.r_{0}r_{-1}r_{-2}\ldots ,\;0.r_{1}r_{2}r_{3}\ldots ).$$

However, the symbolic representation (6) will be more convenient.

An **initial value formulation** determines the entire trajectory of the model Universe from two objects: (1) an initial distribution over the joint microstate of the systems, and (2) the event dynamics, which consist of deterministic state transformations.

The coarse-grained variables are initialized to a given distribution $\mathrm{Pr}(s_{0,\;U})$, on which we place no restrictions. Meanwhile, the microvariables are initialized to be jointly independent (of each other and the coarse-grained variables), and uniformly distributed, taking each value in $Z_{m}$ with equal probability 1/m.

All that remains is to specify the dynamics at each event *t*. It consists of two stages. The first stage is a shift transformation on one system i∈e(t); for concreteness, we let *i* be the least element of e(t). All of system *i*’s microvariables $r_{k}$ shift one position to the left.

The second stage is a bijective transformation

$$f_{t}\left[s_{t-1,\;c(t)}\right]:S_{e(t)}\times Z_{m}\to S_{e(t)}\times Z_{m},$$

that depends on the coarse-grained state $s_{t-1,\;c(t)}$ of the influencing systems c(t). It is applied jointly to the coarse-grained state $s_{t-1,\;e(t)}$ of the evolving systems, and the newly centered microvariable $r_{1}$ of the system i∈e(t):

(8) $$\left(s_{t,\;e(t)},\;r_{1}^{\prime }\right):=f_{t}\left[s_{t-1,\;c(t)}\right]\left(s_{t-1,\;e(t)},\;r_{1}\right).$$

In the case where e(t) contains only one system *i*, its full two-stage transformation is summarized as follows:

$$\begin{array}{cc} (s_{t-1,\;i},\; & \left(\ldots ,\;r_{-2},\;r_{-1},\;r_{0},\;r_{1},\;r_{2},\;\ldots )\right)\\ \overset{\mathrm{shift}}{\to }\;(s_{t-1,\;i},\; & \left(\ldots ,\;r_{-1},\;r_{0},\;r_{1},\;r_{2},\;r_{3},\;\ldots )\right)\\ \overset{\mathrm{transform}}{\to }\;(s_{t,\;i},\; & (\ldots ,\;r_{-1},\;r_{0},\;r_{1}^{\prime },\;r_{2},\;r_{3},\;\ldots )). \end{array}$$

With these deterministic dynamics, the only source of randomness is the initial condition. Since the microvariables are initialized independently and uniformly, we think of each $r_{k}\in Z_{m}$ as a fair *m*-sided die that can be used to emulate a stochastic transition of the coarse-grained variables. As a result, when we ignore all of the $r_{k}$, the coarse-grained variables’ trajectory is given by the persistent causal model (2), with the forward-time transition probabilities

(9) $$\mathrm{Pr}\left(s_{t,\;e(t)}|s_{t-1,\;e(t)},\;s_{t-1,\;c(t)}\right)=\frac{1}{m}\left|\left\{r_{1}:\;\exists r_{1}^{\prime },\;f_{t}\left[s_{t-1,\;c(t)}\right]\left(s_{t-1,\;e(t)},\;r_{1}\right)=\left(s_{t,\;e(t)},\;r_{1}^{\prime }\right)\right\}\right|.$$

Bijectivity of $f_{t}\left[s_{t-1,\;c(t)}\right]$ implies double stochasticity of the forward mechanism (9).

Conversely, *every* doubly stochastic persistent causal model, whose probabilities are multiples of 1/m, is emulated by a causal multibaker map with a suitable choice of bijections $f_{t}\left[s_{t-1,\;c(t)}\right]$. Indeed, given the desired transition probabilities, we need only assign each pair $s_{t-1,\;e(t)},\;s_{t,\;e(t)}\in S_{e(t)}$ to each other with multiplicity $m\cdot \mathrm{Pr}(s_{t,\;e(t)}|s_{t-1,\;e(t)},\;s_{t-1,\;c(t)})$, for each fixed *t* and $s_{t-1,\;c(t)}$. One way to accomplish this is to fix any total order < on $S_{e(t)}$. Then, for all trajectories s, events *t*, and $r\in Z_{m\cdot \mathrm{Pr}(s_{t,\;e(t)}|s_{t-1,\;e(t)},\;s_{t-1,\;c(t)})}$, let

$$\begin{array}{cc} f_{t}\left[s_{t-1,\;c(t)}\right] & \left(s_{t-1,\;e(t)},\;r+\sum_{s_{t,\;e(t)}^{\prime }<s_{t,\;e(t)}}m\cdot \mathrm{Pr}\left(s_{t,\;e(t)}^{\prime }|s_{t-1,\;e(t)},\;s_{t-1,\;c(t)}\right)\right)\\ := & \left(s_{t,\;e(t)},\;r+\sum_{s_{t-1,\;e(t)}^{\prime }<s_{t-1,\;e(t)}}m\cdot \mathrm{Pr}\left(s_{t,\;e(t)}|s_{t-1,\;e(t)}^{\prime },\;s_{t-1,\;c(t)}\right)\right). \end{array}$$

Double stochasticity (3) ensures that each $f_{t}\left[s_{t-1,\;c(t)}\right]$ is well-defined and bijective. Moreover, the fact that *r* takes on $m\cdot \mathrm{Pr}(s_{t,\;e(t)}|s_{t-1,\;e(t)},\;s_{t-1,\;c(t)})$ values, each realized with probability 1/m, ensures the required transition probabilities (2). The Birkhoff-von Neumann theorem implies that $f_{t}\left[s_{t-1,\;c(t)}\right]$ may even be defined in such a way that $r_{1}^{\prime }=r_{1}$ always holds in (8); however, such a definition would be more complicated [61].

Thus, under mild restrictions on the persistent causal model, we can always produce a multibaker map that emulates it. The restriction that the probabilities have a common denominator *m* can be removed, using techniques from [40] or [33]; we only used it here to take advantage of the simpler *m*-ary shift representation. The other restriction is double stochasticity (3). It comes from the microscopic reversibility of the transformation functions $f_{t}\left[s_{t-1,\;c(t)}\right]$, and is a special case of a stationarity condition that comes from coarse-graining a broader class of measure-preserving transformations [33,40].

The correspondence between fine-grained and coarse-grained views has the additional feature of being *local*: the definition of a fine-grained transformation $f_{t}$ uniquely determines the coarse-grained transition probabilities (9), regardless of any other events. So for example, to construct a time-homogeneous Markov chain with N=1, it suffices to repeat the same microscopic transformation $f_{t}$ at every time step.

This works because the transformation (8) is always applied on a “fresh die” $r_{1}$, which is uniform and independent of the coarse-grained trajectory from initialization up to the present. If instead we view the process in reverse, undoing events in reverse order, then the inverse function $f_{t}^{-1}\left[s_{t,\;c(t)}\right]$ is applied to the possibly correlated pair $\left(s_{t,\;e(t)},\;r_{1}^{\prime }\right)$. As such, the time-reversed transition probabilities cannot be locally derived from $f_{t}$ alone; in general, they depend on the earlier transformations and initial conditions. Rather than follow a locally well-defined statistical law as in (9), the reverse dynamics must “conspire” to return to the initial condition.

**Example 3.**  *To get a fine-grained model of Example 1, let m:=3. For $s_{t,\;i}\in Z$ and $r\in Z_{3}$, define the microscopic transformations*

$$\begin{array}{cc} f_{1}\left(s_{0,\;1},\;r\right) & :=(s_{0,\;1}+r-1,\;r),\\ f_{2}\left[s_{1,\;1}\right]\left(s_{0,\;2},\;r\right) & :=(s_{0,\;2}⊕s_{1,\;1},\;r),\\ f_{3}\left(s_{1,\;1},\;r\right) & :=(s_{1,\;1}+r-1,\;r). \end{array}$$

*The fine-grained trajectory of the two systems is listed in Table 1. Here we see a thermodynamic arrow of time: each step of System 1’s random walk adds an independent random variable (first $r_{1}$, then $r_{2}$), increasing the entropy of its coarse-grained state. We also see an epistemic arrow of time: at times t≥2, System 2 maintains a record of $s_{1,\;1}=r_{1}-1$.*

*Starting from the final state at the bottom row of Table 1, we can follow the trajectory in reverse, applying the inverse transformations*

$$\begin{array}{cc} f_{3}^{-1}\left(s_{3,\;1},\;r\right) & :=(s_{3,\;1}+1-r,\;r),\\ f_{2}^{-1}\left[s_{2,\;1}\right]\left(s_{3,\;2},\;r\right) & :=(s_{3,\;2}⊕s_{2,\;1},\;r),\\ f_{1}^{-1}\left(s_{2,\;1},\;r\right) & :=(s_{2,\;1}+1-r,\;r). \end{array}$$

*In doing so, we would witness two strange “miracles”. First, going from t=3 to t=2, System 1’s random walk happens to converge to the value recorded in System 2, despite the systems not interacting during this time interval. And finally, going from t=1 to t=0, the random walk converges to its starting point, clearing all of its entropy.*

*The miracles are explained by the correlation between the “final conditions”*

$$\begin{array}{cc} s_{3,\;1} & =r_{1}+r_{2}-2,\\ s_{3,\;2} & =r_{1}-1, \end{array}$$

*and the microvariables $\left(r_{1},\;r_{2}\right)$ that control the randomness of the coarse-grained dynamics.*

*If instead we set proper final conditions, in which the microvariables at t=3 are independent of $\left(s_{3,\;1},\;s_{3,\;2}\right)$, then the arrow of time would reverse. To be precise, the coarse-grained behavior would be given by the dual model in Example 2. Table 2 and Table 3 list the fine-grained dual trajectories for both sets of coarse-grained final conditions considered in Example 2.*

*In general, the causal arrow of time is determined not by the t-coordinate, but by the graph’s edges. Table 1, Table 2 and Table 3 are all arranged such that the arrow of time points downward.*

**Table 1:** The trajectory of the systems modeled in Example 3. Each $r_{k}\in Z_{3}$ is independent and uniformly distributed.

Time *t*	State of System 1	State of System 2
0	$\left(0,\;\left(\ldots ,\;r_{0},\;\ldots \right)\right)$	(0,(…))
1	$\left(r_{1}-1,\;\left(\ldots ,\;r_{1},\;\ldots \right)\right)$	(0,(…))
2	$\left(r_{1}-1,\;\left(\ldots ,\;r_{1},\;\ldots \right)\right)$	$\left(r_{1}-1,\;\left(\ldots \right)\right)$
3	$\left(r_{1}\;+\;r_{2}\;-\;2,\;\left(\ldots ,\;r_{2},\;\ldots \right)\right)$	$\left(r_{1}-1,\;\left(\ldots \right)\right)$

**Table 2:** The dual model’s trajectory, when its final condition is set to the initial condition of Table 1. Although the expressions appear different, the symmetry of the events renders this coarse-grained trajectory’s distribution identical to that of Table 1.

Time *t*	State of System 1	State of System 2
3	$\left(0,\;\left(\ldots ,\;r_{0},\;\ldots \right)\right)$	(0,(…))
2	$\left(1-r_{0},\;\left(\ldots ,\;r_{-1},\;\ldots \right)\right)$	(0,(…))
1	$\left(1-r_{0},\;\left(\ldots ,\;r_{-1},\;\ldots \right)\right)$	$\left(1-r_{0},\;\left(\ldots \right)\right)$
0	$\left(2\;-\;r_{0}\;-\;r_{-1},\;\left(\ldots ,\;r_{-2},\;\ldots \right)\right)$	$\left(1-r_{0},\;\left(\ldots \right)\right)$

**Table 3:** The dual model’s trajectory, when the coarse-grained part of its final condition is set to the final distribution of Table 1. The reason it does not simply retrace the trajectory of Table 1, is that the microvariables here are reset to fresh independent values.

Time *t*	State of System 1	State of System 2
3	$\left(r_{1}\;+\;r_{2}\;-\;2,\;\left(\ldots ,\;r_{0},\;\ldots \right)\right)$	$\left(r_{1}-1,\;\left(\ldots \right)\right)$
2	$\left(r_{1}\;+\;r_{2}\;-\;r_{0}\;-\;1,\;\left(\ldots ,\;r_{-1},\;\ldots \right)\right)$	$\left(r_{1}-1,\;\left(\ldots \right)\right)$
1	$\left(r_{1}\;+\;r_{2}\;-\;r_{0}\;-\;1,\;\left(\ldots ,\;r_{-1},\;\ldots \right)\right)$	$\left(r_{1}\;-\;1⊕r_{1}\;+\;r_{2}\;-\;r_{0}\;-\;1,\;\left(\ldots \right)\right)$
0	$\left(r_{1}\;+\;r_{2}\;-\;r_{0}\;-\;r_{-1},\left(\ldots ,r_{-2},\ldots \right)\right)$	$\left(r_{1}\;-\;1⊕r_{1}\;+\;r_{2}\;-\;r_{0}\;-\;1,\;\left(\ldots \right)\right)$

**Figure 3:**
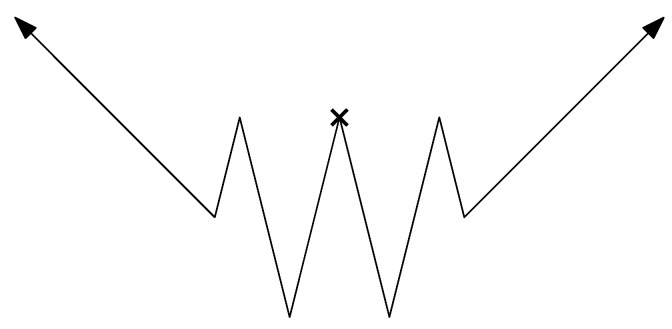
A conceptual visualization of the arrow of time. The horizontal axis represents the time coordinate, while the vertical axis represents entropy. If the state distribution of a multibaker map is absolutely continuous at some initialization time (the small cross), then some duration around it (the wavy central region) may behave ambiguously, but eventually the arrow of time points consistently away to ±∞. The dynamics of the left region are dual to those of the right region.

We conclude that the causal arrow of time always points away from the initialization time, at which the microvariables are independent and uniform. In fact, the initialization need not be so strict: provided that the initial distribution is reasonably “smooth” on its phase space geometry (7), the microvariables $r_{k}$ become increasingly uniform as k→±∞. After sufficiently many iterations of the shift transformation (or its inverse), it follows that the coarse-grained dynamics become indistinguishable from the case of uniform microvariable initialization; see [33] for a rigorous proof. Therefore, as depicted in Figure 3, the arrow of time points away from the initialization time toward ±∞, except that its direction may temporarily be ambiguous near initialization.

One may speculate about whether the real-world Past Hypothesis (at the Big Bang) can be expressed in similar terms. Instead of the shift transformation, a chaotic Hamiltonian evolution would give the phase space distribution an increasingly fine filamentary structure, until it effectively “looks uniform” on a larger region of phase space [2]. Of course, this argument applies equally well in reverse; Boyle et al. [62,63] suggest that a CPT-inverted dual dynamics may prevail before the Big Bang.

## 4. The Arrows of Time

The causal multibaker maps exhibit some time asymmetries that are analogous to those we see in the real Universe. As such, these maps can be said to model the arrows of time in a mathematically precise and tractable way. We now examine these asymmetries.

### 4.1. The Causal Arrow of Time

Despite their deterministic and reversible fine-grained evolution, we saw that causal multibaker maps with suitable initial conditions exhibit asymmetric coarse-grained behavior. This behavior is exactly described by the persistent causal model (2). It satisfies the graph’s Markov property, so that, for example, every correlated pair of events has a common cause from an earlier time. Moreover, the coarse-grained mechanisms are local functions of the corresponding fine-grained transformations.

To be precise, when the model represents the Universe as a whole, then it can only be interpreted as a Bayesian model, not a causal one. The do operator, required for causal semantics, amounts to a modification of the dynamics, which is not possible from a global point of view. On the other hand, we gave plausible arguments for the do operator’s practical relevance, to model exogenous influences on subsystems that occur repeatedly in the Universe.

Indeed, Bell [5,6] observed that the validity of the scientific method rests on an experimenter’s ability to independently choose or randomize control variables in a repeated experiment. Reusing the causal network in Figure 1, we may interpret System 1 as an experimenter, who sets a control variable in System 2. The interaction mechanism $\mathrm{Pr}(s_{2,\;2}|s_{1,\;1},\;s_{0,\;2})$ is determined by local considerations, represented by the fine-grained transformation $f_{2}$. In particular, $S_{2,\;2}$ can be randomized independently of past states, regardless of any other mechanisms in the model. The reverse is not possible: depending on other mechanisms, the future coarse-grained state of the Universe may in general correlate with $S_{0,\;2}$ and/or $S_{2,\;2}$.

As an additional remark, if we want to define interventions and counterfactuals on a causal multibaker map, there is no conflict between Lewis [20]’s closest-world semantics and Pearl [9]’s structural semantics. Lewis’ version of the counterfactual $S^{do(S_{t,\;i}=x)}$ is defined as the Universe closest to S that satisfies $S_{t,\;i}=x$. If we define distance by the number of altered fine-grained mechanisms, breaking ties by the number of altered state values, then the closest Universe satisfying $S_{t,\;i}=x$ is one which alters only the mechanism $f_{t}$ with respect to system *i*. Thus, we recover Pearl’s semantics.

### 4.2. The Thermodynamic Arrow of Time

The second law of thermodynamics can be derived as a consequence of the Markov property and double stochasticity. In the special case of Markov chains, the second law is a very well-known mathematical theorem.

**Theorem 1**  (Second law of thermodynamics for Markov chains)**.** *Consider any Markov chain, given by (2) with N=1. Suppose 0≤t≤u≤T, and that all events v=t+1,…,u are doubly stochastic. Then,*

$$H\left(S_{t,\;1}\right)\leq H\left(S_{u,\;1}\right).$$

**Proof.**  See Section 4.4 in Cover and Thomas [54]. □

Theorem 1 provides the most well-understood arrow of time. It implies that all entropy-increasing processes are irreversible. Perhaps it is a bit ironic that, for the multibaker maps, the irreversible growth of entropy is a consequence of double stochasticity, which in turn is a consequence of $f_{t}$’s reversibility.

By conditioning on any exogenous influences, we extend Theorem 1 to non-isolated systems.

**Theorem 2**  (Second law of thermodynamics for causal models)**.** *Consider any persistent causal model (2), and 0≤t≤u≤T. Let A,B⊂U be composite systems with the property that for all v=t+1,…,u:*

- *Either e(v)⊂A, or e(v)⊂B, or e(v)⊂U∖(A∪B).*
- *If e(v)⊂A, then c(v)⊂A∪B and the event v is doubly stochastic.*
- *If e(v)⊂B, then c(v)⊂B.*

*Then,*

(10) $$H\left(S_{t,\;A}|S_{t,\;B}\right)\leq H\left(S_{u,\;A}|S_{u,\;B}\right).$$

**Proof.**  Removing elements from *A* that are shared with *B* does not change the conditional entropy; hence, we lose no generality in assuming that *A* and *B* are disjoint. By mathematical induction, it suffices to prove (10) over the span of a single event u=t+1. Depending on the systems e(u) that it acts on, there are three cases to consider.

When e(u)⊂U∖(A∪B), the composite systems *A* and *B* are unchanged, so (10) holds with equality.

When e(u)⊂A, we have $S_{t,\;B}=S_{u,\;B}$. Moreover, c(v)⊂A∪B, so the composite system A∪B undergoes doubly stochastic transition probabilities with no outside influence. Treating A∪B as one system, Theorem 1 implies $H\left(S_{t,\;A\cup B}\right)\leq H\left(S_{u,\;A\cup B}\right)$. Therefore,

$$\begin{array}{cc} H(S_{t,\;A}|S_{t,\;B})\; & =H\left(S_{t,\;A\cup B}\right)-H\left(S_{t,\;B}\right)\\ & \leq H\left(S_{u,\;A\cup B}\right)-H\left(S_{u,\;B}\right)\\ & =H(S_{u,\;A}|S_{u,\;B}). \end{array}$$

Finally, when e(u)⊂B, we have c(u)⊂B and $S_{t,\;A}=S_{u,\;A}$. By the d-separation criterion, the latter variable is conditionally independent of $S_{u,\;B}$, given $S_{t,\;B}$, so the data processing inequality ([54] Thm 2.8.1) implies $I\left(S_{t,\;A}:S_{t,\;B}\right)\geq I\left(S_{u,\;A}:S_{u,\;B}\right)$. Therefore,

$$\begin{array}{cc} H(S_{t,\;A}|S_{t,\;B})\; & =H\left(S_{t,\;A}\right)-I\left(S_{t,\;A}:S_{t,\;B}\right)\\ & \leq H\left(S_{u,\;A}\right)-I\left(S_{u,\;A}:S_{u,\;B}\right)\\ & =H(S_{u,\;A}|S_{u,\;B}). \end{array}$$

□

Since the conditional entropy in question can be expressed as

$$H\left(S_{t,\;A}|S_{t,\;B}\right)=H\left(S_{t,\;A}\right)-I\left(S_{t,\;A}:S_{t,\;B}\right),$$

Theorem 2 allows the composite system *A*’s entropy to decrease, provided that the mutual information term also decreases. The influence of a “Maxwell’s demon” *B*, with knowledge (i.e., mutual information) about *A*’s state, can therefore decrease *A*’s entropy [46,64].

### 4.3. The Epistemic Arrow of Time

“The epistemic arrow of time is the fact that our knowledge of the past seems to be both of a different kind and more detailed than our knowledge of the future.” - Wolpert and Kipper [12]

Consider the difference between weather forecasts (of the future) and historical weather records (of the past). Weather forecasts are derived from computationally intensive simulations seeded with precise global measurements, and become unreliable beyond about a week. In contrast, precise records can be produced with minimal computational effort or meteorological expertise, and then maintained for millennia.

Wolpert and Kipper [11,12] distinguish memories that are “Type-2” or “computer-type”, from those that are “Type-3” or “photograph-type”. A computer-type memory is not considered a record. It uses information about the present state and dynamics of another system, to simulate its evolution very precisely, either backward or forward in time. In this manner, computer-type memories can produce predictions about both the past and future.

In order for the simulation to be accurate, computer-type memories require knowledge of the other system’s dynamics, including its interactions with additional systems, which may in turn interact with more systems, and so on. Moreover, when the dynamics are chaotic, the simulation must start from extremely fine-grained knowledge of the state, which is simply not available in our coarse-grained view.

In contrast, photograph-type memories do serve as records. Slightly adapting the definition of Mlodinow and Brun [14], we consider records to be systems, whose coarse-grained state at some designated time $t_{read}$, is a non-constant function of another system’s coarse-grained state, at some other time $t_{event}$, *regardless of the latter system’s dynamics*. In Example 1, System 2 fits this definition with $t_{event}=1$ and $t_{read}\in \left\{2,3\right\}$. System 2 does not simulate System 1’s dynamics; instead, it stores the event state directly. As a result, even if we were to change the initial state and dynamics of System 1 entirely, System 2 would still successfully record the event $S_{1,\;1}$.

Records can only be of the past, as any attempt to record a future event would be foiled by some choice of the dynamics leading up to that event. Imagine extending the network in Figure 1 to much larger times, with occasional interactions between the two systems. Suppose we control the mechanisms that initialize and evolve System 2 (the memory), but System 1 always takes random steps. Then, $S_{2,\;u}$ cannot know the state of System 1 after the most recent interaction time t<u. Indeed, by the d-separation criterion, conditional on $S_{1,\;t}$, any subsequent random steps taken by System 1 are independent of $S_{2,\;u}$.

Note that our explanation makes no use of the thermodynamic arrow. In contrast, Wolpert and Kipper [11,12] argue that photograph-type memories require an entropy-producing initialization step. While entropy is produced in almost all real-life examples, in some sense this is a trivial observation: a Universe with a thermodynamic arrow will certainly never lose entropy, and it is rather difficult to keep entropy exactly constant. This applies not only to memories, but to all physical processes. Nonetheless, Bennett [65] shows that in principle, memory initialization can be performed in a thermodynamically reversible (entropy-preserving) manner.

Another way to see that the classical second law of thermodynamics (Theorem 1) cannot possibly tell the whole story is to consider the joint entropy of two non-interacting systems (i.e., independent Markov chains)

$$H\left(S_{t,\;\{1,2\}}\right)=H\left(S_{t,\;1}\right)+H\left(S_{t,\;2}\right)-I\left(S_{t,\;1}:S_{t,\;2}\right).$$

Theorem 1 applies to each of the systems as well as to their union. Hence, the total entropy $H(S_{t,\;\{1,2\}})$, as well as each of the terms $H(S_{t,\;1})$ and $H(S_{t,\;2})$, are non-decreasing. This does not rule out the possibility of the systems spontaneously becoming correlated: the second law is consistent with an increase in $I(S_{t,\;1}:S_{t,\;2})$, provided that the other terms increase as well to compensate. The data processing inequality ([54] Theorem 2.8.1) forbids this as a separate consequence, not of Theorem 1, but of the causal assumptions.

It therefore seems reasonable to think of the arrows of time as fundamentally originating from causality, rather than from the second law of thermodynamics.

### 4.4. The Evolutionary Arrow of Time

Darwinian evolution is a process by which creatures adapt to their environment, as a result of repeated exposure to its selective pressures over the span of many generations. While environments can change, the homogeneity of the laws of physics across space and time provide some degree of consistency.

As we saw previously, for a causal multibaker map with a time-independent law $f_{t}$, the coarse-grained forward-time probabilities are also time-independent, while the reverse-time probabilities generally are not. If we try to cheat the arrow of time by hardcoding advanced creatures into the initial condition, we must ask: what are these creatures adapted to? They cannot have adapted to the underlying law $f_{t}$, because the coarse-grained reverse-time trajectory would always converge to the initial condition, regardless of $f_{t}$.

In contrast, we saw that the coarse-grained *forward*-time probabilities are locally determined by $f_{t}$. They determine the statistical properties of emergent processes, such as genetic mutation, survival, and reproduction, which in turn determine the natural selection gradient. If the law $f_{t}$ is applied consistently across a large environment over many generations, then the gene pool evolves along that gradient.

### 4.5. The Agential/Volitional Arrow of Time

If causal multibaker maps could support the evolution of intelligent agents, then perhaps we should not be so surprised if those agents come to regard the past as “fixed”, and the future as “open” to influence from their actions. When discussing the epistemic arrow, we saw that there exist mechanisms which make reliable records of past events; no action from an agent can invalidate a record, short of vandalizing the record itself. In contrast, we saw that no such records exist for future events.

The evolutionary arrow yields additional insights. In the context of Darwinian evolution, the interventions and counterfactuals corresponding to our alternative actions take on a very real meaning: they model competing agents who respond to the same situation with different algorithms. Some of these agents will be more successful than others. An intelligence with the ability to imagine itself in the role of every possible agent, and then acting as the most successful one, will enjoy the maximum survival advantage. Therefore, natural selection increases the prevalence of intelligences that behave this way.

Rovelli [4,18] presents a different account of the agential arrow, in which decisions are considered to be random and entropy-producing. Our approach is closer to that of Rehn [60]: we do not require decisions to be random, but instead view them as outputs of an algorithm that computes estimated outcomes for many different actions.

## 5. Discussion

Historically, the lack of a mathematically precise model for emergent time-reversal asymmetries posed a major obstacle to their detailed study. While the original multibaker maps provided useful models for thermodynamics [39,40], they lacked the causal interactions responsible for the epistemic, evolutionary, and agential arrows of time. Meanwhile, Pearlean causal models successfully captured these asymmetries [9], but provided no connection to reversible dynamical laws.

Bridging these ideas together, our causal multibaker maps coarse-grain into persistent causal models, offering a tractable, precise framework that can be configured for arbitrary causal interactions in discrete time and space. Follow-up work can split in two main directions.

The first is to study the properties of persistent causal models and use them to model additional phenomena. In particular, the evolutionary and agential arrows should be investigated in much greater depth. Papadopoulos et al. [66] find that machine prediction performs better in a forward-time direction, suggesting that learning algorithms might take advantage of the causal structure (1). Causal modeling may also yield insights on the thermodynamics of biochemical and computational systems [47,67].

The second direction is to investigate how the emergence of time asymmetries in the real Universe resembles or differs from their emergence in causal multibaker maps. Our maps are highly stylized, ignoring many important aspects of real physics. For example, spacetime has a manifold structure, whose most obvious asymmetry is the fact that it is expanding; this is the famous **cosmological arrow of time** [68]. Quantum extensions of causal modeling concepts are another highly active area of research, that will likely require reevaluating our classical intuitions [69,70,71,72,73,74,75]. Since the discrete analogue of a field theory is a lattice or cellular automaton, it would also be worth exploring how causal sparsity arises in such models [33,52,76]

A more subtle difference is that causal multibaker maps ensure the Markov property by conveniently shifting their microvariables $r_{k}$. In the real Universe, it is much less clear whether and how the Markov property arises [34,35,36,37,38]. In place of our microvariables, chaos or quantum decoherence might play a role in providing independent sources of randomness [77,78,79].

The epistemic arrow exposes a deeper issue: records only give us information if we know how to interpret them. To do so, we require prior knowledge of the memory’s mechanism and initial state. If we feign ignorance and assume a uniform Bayesian prior on the state of the Universe, then we effectively treat the Universe as if it were at heat death. By the second law of thermodynamics, we can never return to a state of non-ignorance, as every observation would be suspected of being a random “Boltzmann brain” fluctuation [33,80,81]. As explained by Wolpert and Kipper [12], this issue is closely related to formal impossibility results in the theory of inductive inference [82,83].

The question becomes: how should a living being embedded inside a causal multibaker map infer non-uniform probabilities such as (2)? Scharnhorst et al. [84] suggest that we should condition on some trustworthy physics knowledge: specifically, the initial and current entropy and dynamics of the Universe. Another approach, based on Occam’s razor, uses algorithmic information theory to define a simplicity prior [85,86,87,88]. Algorithmic information theory might add more nuance to our understanding of the arrows of time, as it defines probability-free notions of entropy and causality [89,90,91,92], as well as alternative measures of complexity such as Bennett’s logical depth [93,94].

Finally, we argued that evolution favors agents who reason in terms of forward-time causal interventions. However, in settings where multiple agents must reason about each other’s behavior, Yudkowsky, Soares, and Levinstein [95,96] argue that it is sometimes advantageous to choose actions as if they could affect the physical past. In light of ongoing advances in the psychology and neuroscience of time perception [97,98,99,100], one could try to study whether human psychology includes such exceptions. As human and artificial intelligence continues to advance and collaborate at larger scales, it becomes increasingly important to clarify the causal foundations of inference and decision theory.

## Data Availability

The original contributions presented in the study are included in the article, further inquiries can be directed to the corresponding author.
